# Risk factors for mortality and multiresistant bacteria in bloodstream infections acquired in a private hospital intensive care unit

**DOI:** 10.1186/2197-425X-3-S1-A888

**Published:** 2015-10-01

**Authors:** JM Bonell Goytisolo, I Jara Zozaya, R Vento Rehues, MT Millán Guilarte, P Mas Morey, FJ Montero Clavero

**Affiliations:** Intensive Care Unit, Hospital Quirón Palmaplanas, Palma de Mallorca, Spain; Hospital Pharmacy, Hospital Quirón Palmaplanas, Palma de Mallorca, Spain

## Introduction

Nosocomial infections are a huge health problem worldwide, among them bacteremia are one of the most relevant in terms of morbidity and costs.

## Objective

To study whether multiresistant bacterial (MRB) etiology and mortality of ICU-acquired bacteremia are associated with epidemiological and clinical factors of the episode and patient.

## Methods

Retrospective, and cohort in the last 12 months, study of 70 consecutive episodes of bacteremia between 2011 and 2014 in a polyvalent 15 bed unit. Several items related to patient and episode are analyzed.

## Results

Sixty-six different patients, median age 69 years (25-87). Most common origins were central catheter in 22 (31%), lung in 15 (21%) and abdomen in 10 (14%). Gram-negative bacteria alone caused 32 cases (46%, Pseudomonas and Enterobacter were predominant, with 15 cases of MRB), Gram-positive 29 (41%, coagulase-negative staphylococci was the most common), fungi in 9% and mixed origin in 4%. 36 patients (55%) had been previously treated with broad spectrum antibiotics; 40 patients (61%) were under mechanical ventilation, 52% were surgical, 23% with parenteral nutrition and 12% under renal replacement therapy. 36% of patients suffered from shock in the first 48 h of admission in ICU and 30% had neoplasm or other inmunosupresive situation at the admission. 14% of episodes had another previous infection caused by the same bacteria. In 26% of cases clinical manifestation of bacteremia was septic shock.

The presence of MRB had significant association with pulmonary origin (OR 4.8; CI 95% 1.3 - 17; p = 0.02), mechanical ventilation (OR 4.7; CI 95% 1.2 - 18.5; p = 0.03) and a previous infection by the same germ (OR 11.7; CI 95% 2.6 - 53; p < 0.01). Previous broad spectrum antibiotic therapy was related but was not significant.

Hospital mortality was 27%, we found association with previous treatment with piperacilin-tazobactam (OR 3.7; CI 95% 1.1 - 12.9; p = 0.04); other factors such as parenteral nutrition and septic shock complicated bacteremia did not reach statistical signification.

## Conclusions

Acquired bacteremia in our unit is an heterogeneous complication attending to their origin and etiology. Risk factors for MRB were pulmonary source, mechanical ventilation and previous infection by the same bacteria. Mortality in our study was related to previous exposure to piperacilin-tazobactam, however we think that more cohort studies with large number of patients are required.
